# Neutrophil extracellular traps degrade fibronectin in a rat model of bronchopulmonary dysplasia induced by perinatal exposure to lipopolysaccharide

**DOI:** 10.1111/jcmm.15842

**Published:** 2020-10-23

**Authors:** Junjie Xu, Xiaonan Mao, Rui Jin, Jiao Yin, Keyu Lu, Yan Guo, Mingshun Zhang, Rui Cheng

**Affiliations:** ^1^ Department of Neonatal Medical Center Children’s Hospital of Nanjing Medical University Nanjing China; ^2^ NHC Key Laboratory of Antibody Technique Department of Immunology Nanjing Medical University Nanjing China

Dear Editor,

Over the 50 years since its first description, bronchopulmonary dysplasia (BPD) has gained major advances in therapy, including antenatal corticosteroids, continuous positive airway pressure and surfactant replacement.^1^ However, BPD is still the most common chronic respiratory morbidity in preterm infants, and the challenge of exploring unmasked culprits in BPD pathogenesis remains unresolved. Neutrophils are abundant leucocytes in acute inflammation. Peripheral blood neutrophil counts are increased in infants with moderate‐severe BPD, and the prolonged influx of neutrophils into the lung is associated with BPD severity,^2^ suggesting that neutrophils may contribute to BPD.

As a major component of pulmonary ECM, expression of fibronectin coincides with lung development,^3^ especially fibronectin is indispensable for branch morphogenesis in murine lung explants.^4^ Moreover, fibronectin expression is directly linked with alveolar septation, a key step in alveologenesis.^5^ The differentiation of alveolar epithelial cells, the major constituents of the alveolus in the developing lung, is regulated by fibronectin.^6^ As inadequate fibronectin impairs the airway epithelial branching morphogenesis,^7^ we hypothesize that fibronectin deficiency may retard lung branching development, thereby promoting BPD initiation.

Neutrophil extracellular traps (NETs), first described in the immune defence against bacterial infection, have been linked to diverse pulmonary diseases.^8^ Recently, it is reported that neutrophil elastase from neutrophils exosome destructs extracellular matrix (ECM) and induces BPD‐like disease in mice.^9^ NETs are quite different from exosomes. However, NETs also contain various proteases. To the best of our knowledge, the roles of NETs on the fibronectin expression have not been investigated in BPD pathogenesis.

The rat model of BPD was established by intra‐amniotic LPS injection in pregnant rats (see Appendix S1 in Supporting Informaion). Compared with the control group, the number of alveoli in the BPD group was significantly reduced. By contrast, mean linear intercept in the developing lung from LPS control was significantly increased (Figure 1A). As fibronectin played key roles in branching morphogenesis and alveolar epithelial cells differentiation, we found that pulmonary fibronectin 1st day post‐birth (P1) was significantly lower in LPS group the than that in PBS control group (Figure 1B,C). Expression of fibronectin from P3 lung tissues from LPS or PBS group, however, was comparable. Similar observation was recorded for P7 lung tissues. LPS down‐regulated fibronectin and distal airway branching in the foetal lung.^10^ To explore whether LPS directly decreased fibronectin in alveolar epithelial cells, we treated MLE‐12 cells (alveolar epithelial cell line) or primary alveolar epithelial cells with LPS. Indeed, LPS directly reduced fibronectin in both MLE‐12 cells (Figure 1D,E) and primary alveolar epithelial cells (Figure 1F). The TGF‐β/Smad signalling pathway was essential for fibronectin expression in bronchial epithelial cells. Similarly, Smad‐3 deficiency retarded lung alveolarization.^11^ As expected, LPS decreased TGF‐β and inactivated Smad‐3 on alveolar epithelial cells (Figure 1G). By contrast, Smad‐3 inhibitor (SIS3) rescued the expression of fibronectin on epithelial cells (Figure 1H). TLR‐4 inhibitor (TAK‐242), which blocked the roles of LPS, also reversed the reduction in fibronectin on epithelial cells (Figure 1I). Collectively, these results implied that LPS bound to TLR4 down‐regulated the ECM protein fibronectin on alveolar epithelial cells, promoting the progression of BPD.

**Figure 1:**
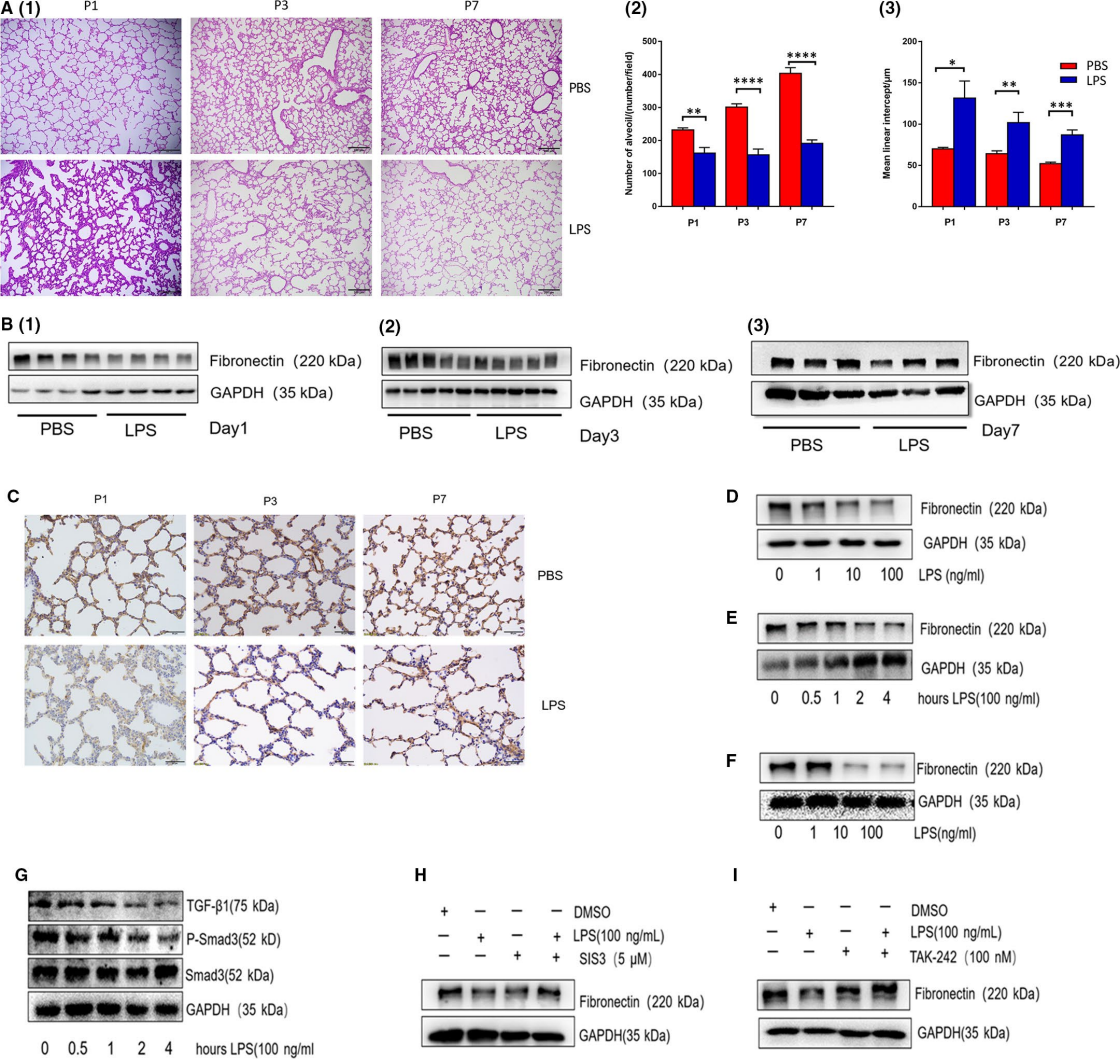
LPS decreased fibronectin in a rat model of BPD (A) E16.5 pregnant rats were treated with LPS or saline via intra‐amniotic injection, five pregnant rats in each group. Newborn puppies were sacrificed 1st day post‐birth (P1, five puppies were from PBS group, five from LPS group), P3 (seven puppies from PBS group, six from LPS group) and P7 (six puppies from PBS group, seven from LPS group). (1) Lung morphometry was analysed by haematoxylin and eosin (H&E) staining of tissue samples. Bar = 200 μm. (2) LPS decreased the number of alveoli compared with the injection of saline. (3) LPS increased the mean linear intercept compared with the injection of saline. B, Pulmonary expression of fibronectin was analysed by Western blotting. Expression of fibronectin in LPS group was lower at P1 compared with that in the control group; there was no significant difference at P3 and P7. C, Fibronectin expression was evaluated by immunohistochemical analysis. Bar = 50 μm. 3‐5 newborn puppies from each group were randomly selected for analysis. D, Fibronectin was decreased in a dose‐dependent manner in MLE‐12 cells stimulated by LPS for 24 h. E, Fibronectin was decreased in a time‐dependent manner in MLE‐12 cells stimulated by 100 ng/mL LPS. F, Fibronectin was decreased in primary alveolar epithelial cells stimulated by LPS for 24 h. G, LPS inactivated TGF‐β1 and Smad‐3 in MLE‐12 cells. H, A Smad‐3 inhibitor (SIS3) restored the expression of fibronectin in alveolar epithelial cells. I, A TLR4 inhibitor (TAK‐242) restored the expression of fibronectin in alveolar epithelial cells. *P < .05; **P < .01; ***P < .0001; ****P < .00001

Besides the direct effects, LPS was a potent inducer of NETs. We speculated that LPS may trigger the formation of NETs in the rat model of BPD. In the lung tissues from LPS‐treated rats, myeloperoxidase (MPO) and citrullinated histone 3 (H3cit) double‐stained structures were recorded in the alveoli (Figure 2A), indicating the formation of NETs. NETs, decorated with protein enzymes in the DNA fibrous backbones, are potential to cleave ECM components.^12^ To investigate whether NETs play roles in the fibronectin expression of alveolar epithelial cells, we stimulated MLE‐12 cells with purified NETs in vitro. NETs decreased fibronectin on alveolar epithelial cells in a time‐ and dose‐dependent manner (Figure 2B,C). DNase, destroying the DNA backbones of NETs (Figure 2D), blocked the reduction in fibronectin induced by NETs (Figure 2E), further demonstrating that NETs decreased fibronectin expression in alveolar epithelial cells. NETs cleaved ECM via elastase and MMP‐9.^12^ We asked whether MMP‐9 was involved in the fibronectin reduction from NETs‐treated alveolar epithelial cells. As shown in Figure 2F, similar to DNase, MMP‐9 inhibitor recuperated the expression of fibronectin, suggesting that NETs reduced fibronectin expression from alveolar epithelial cells via MMP‐9. These results suggested that NETs decreased the expression of fibronectin on alveolar epithelial cells, which was dependent on MMP‐9 and the TGF‐β1/Smad‐3 pathway.

**Figure 2:**
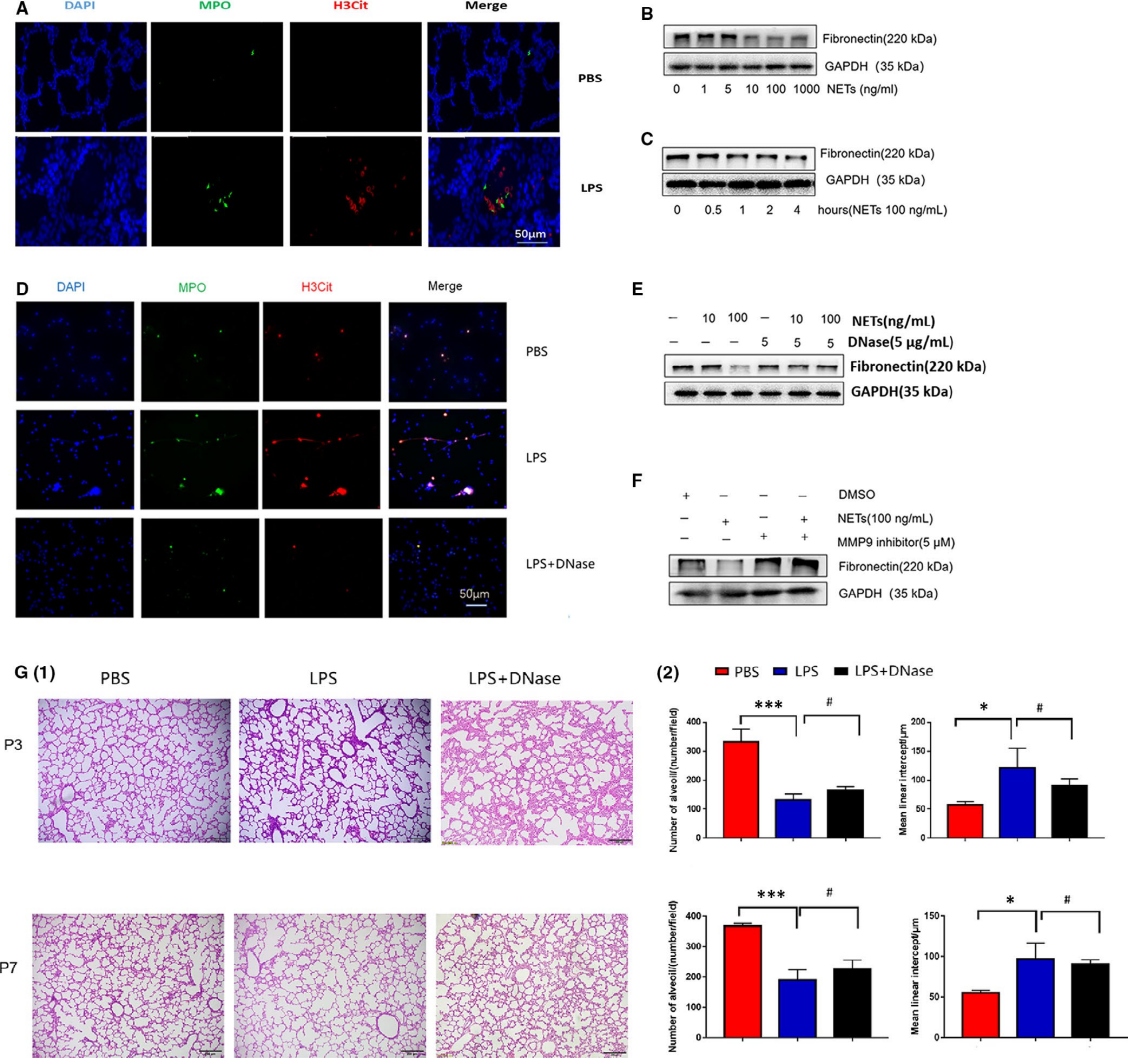
NETs decreased fibronectin in alveolar epithelial cells via MMP‐9. A, Immunofluorescence staining showed NETs formation in lung tissues from LPS‐treated rats (P1); DAPI, nucleus, blue; MPO, red; H3cit, green; bar = 50 μm; (B) Fibronectin was decreased in a dose‐dependent manner in MLE‐12 cells stimulated by NETs for 24 h. C, Fibronectin was decreased in a time‐dependent manner in MLE‐12 cells stimulated by 100 ng/mL NETs. D, DNase destructed NETs induced by LPS in vitro. E, DNase restored the expression of fibronectin in alveolar epithelial cells. F, MMP‐9 inhibitor restored the expression of fibronectin in alveolar epithelial cells. G, DNase did not improve lung development in the rat model of BPD. (1) Lung morphometry was analysed by haematoxylin and eosin (H&E) staining of tissue samples from pups at P3 and P7. Bar = 200 μm. (2) The number of alveoli and mean linear intercept were comparable in the rat model of BPD treated with or without DNase. n = 3 per group *P < .05; ***P < .011; #*P* > .05

As DNase blocked the role of NETs in the reduction in fibronectin on alveolar epithelial cells in vitro, we speculated that rats with BPD may benefit from DNase therapy. However, the number of alveoli and the mean linear intercept were comparable in either vehicle group or DNase group in the rat model of BPD (Figure 2G), implying that DNase unexpectedly failed to alleviate BPD disease severity. DNase was effective to degrade NETs. However, roles of DNase in blocking NEs function were arguable; DNase degraded DNA backbone of NETs but not essentially deactivated protein enzymes on NETs. Instead, NETs‐associated bioactive enzymes may still be attached into the tissues treated with DNase.^13^ Moreover, DNase even increases enzymes activity, including elastase and other enzymes, in the NETs from cystic fibrosis.^14^ In addition, DNase may be ineffective to neutralize the enzymes activity from exosomes in the NETs preparation. Elastase is directly associated with BPD onset.^9^ By contrast, elastase inhibitor enables lung growth in BPD.^15^ Therefore, DNase in the roles of NETs and BPD was a double‐edged sword. Ongoing efforts were required to explore the precise roles of DNase and NETs in the pathogenesis of BPD.

Taken together, the present study revealed that the pulmonary ECM protein fibronectin was decreased in a rat model of BPD induced by LPS exposure. LPS degraded fibronectin on the alveolar epithelial cells via the TGF‐β/Smad pathway and promoted the formation of NETs in the alveolus. NETs cleaved fibronectin via MMP‐9, which further decomposed the extracellular matrix in the alveolus. DNase degraded NETs but fail to improve disease severity in the rat model of BPD. Our study shed new light on BPD pathogenesis.

## CONFLICT OF INTEREST

The authors declare that the research was conducted in the absence of any commercial or financial relationships that could be construed as a potential conflict of interest.

## AUTHOR CONTRIBUTIONS

**Junjie Xu:** Formal analysis (lead); Investigation (lead); Methodology (lead); Resources (equal); Writing‐original draft (supporting). **Xiaonan Mao:** Investigation (equal); Methodology (equal). **Rui Jin:** Investigation (supporting); Methodology (supporting); Validation (equal). **Jiao Yin:** Investigation (supporting); Methodology (supporting). **Keyu Lu:** Formal analysis (supporting); Investigation (supporting); Methodology (supporting). **Yan Guo:** Conceptualization (supporting); Formal analysis (supporting). **Mingshun Zhang:** Conceptualization (equal); Data curation (equal); Formal analysis (equal); Funding acquisition (equal); Investigation (equal); Project administration (equal); Supervision (equal); Writing‐original draft (equal); Writing‐review & editing (equal). **Rui Cheng:** Conceptualization (equal); Funding acquisition (equal); Project administration (equal); Writing‐review & editing (equal).

## DATA AVAILABILITY STATEMENT

The data that support the findings of this study are available from the corresponding author upon reasonable request.

## Supporting information

Appendix S1
