# Supplementary material for: Neutrophil extracellular traps degrade fibronectin in a rat model of bronchopulmonary dysplasia induced by perinatal exposure to lipopolysaccharide
Source: J Cell Mol Med. 2020 Oct 23;24(24):14645–9. doi: 10.1111/jcmm.15842 (PMC7754010; doi:10.1111/jcmm.15842)
Supplement: Supplementary file 1 — Appendix S1 [file JCMM-24-14645-s001.docx]

**Supplementary materials**

**Materials and methods**

**Experimental animals**

Timed-pregnancy Sprague-Dawley (SD, 400-500g) rats and female C57BL/6 mice (6~8 weeks old) were purchased from the Animal Center of Nanjing Medical University. Animals were housed in a temperature- and humidity-controlled environment and under specific pathogen-free conditions and were granted free access to food and water. Animal experiments were approved by the Laboratory Animal Ethics Committee of Nanjing Medical University (IRB: IEC of Children's Hospital affiliated with Nanjing Medical University, permit number: IACUC-1804010).

**Reagents and antibodies**

LPS (*Escherichia coli* serotype O55:B5), anti-fibronectin (F3648), and DNase I (DN25) were purchased from Sigma-Aldrich (St. Louis, MO). Anti-α-SMA (ab21027), anti-TGF-β1 (ab170874), anti-histone H3 (citrulline R2+R8+R17) (ab5103), anti-myeloperoxidase (MPO, ab90810), and MMP-9 inhibitor (ab142180) were purchased from Abcam (Cambridge, UK). The anti-β-actin (4970L), anti-GAPDH (5174S), anti-Smad3 (9523S), and anti-phospho-Smad3 (9520S) antibodies were purchased from Cell Signaling Technology (Beverly, MA). The TLR4 inhibitor (TAK-242) was purchased from MCE (Shanghai, China). The Smad3 inhibitor (SIS3, S7959) was purchased from Selleck (Shanghai, China). Alexa-Fluor-555-conjugated goat anti-rabbit antibody (1:500, A21428) was purchased from Thermo Scientific (USA). PE-anti-mouse CD284 (TLR4) (145404) was purchased from Biolegend (San Diego, USA). The goat anti-mouse IgG (H+L) cross-adsorbed secondary antibody, Alexa-Fluor-647-conjugated goat anti-mouse antibody (1:500, A21235), was purchased from Life Technology (Basingstoke, UK).

**Bronchopulmonary dysplasia model and DNase treatment**

For the establishment of chorioamnionitis, 10 pregnant SD rats 16.5 days postcoitum (embryos 16.5, E16.5) were randomized into two groups ^1^: (1) the intra-amniotic LPS injection group (0.2 μg/μl, 5 μl, solubilized in saline) and (2) the intra-amniotic saline injection group (5 μl normal saline). Rats were anesthetized by 10% chloral hydrate (3.5-4.0 ml/kg body wt, intraperitoneally), and a 3-cm midline abdominal incision allowed externalization of the uterine horns. A ﬁne-tipped pulled glass pipette was used for direct intra-amniotic injection of 5 μl of sterile, endotoxin-free saline or LPS into the amniotic sac of each fetus. After internalization of the uterus, the abdominal wall was sutured in two layers. Rats were returned to their cages and to free access to food and water. After delivery, pups were kept with their mother and euthanized at postnatal day 1 (P1), P3, and P7 for further investigation.

To treat BPD rats, the experimental group received twice-daily DNase I, and the control group received twice-daily saline. The treatment was performed in the morning and afternoon by aerosolization. In the experimental group, the rats birthed within 24 hours were treated with 5 mL DNase (5 mg/kg) that was dissolved in 15 mL saline and aerosolized using a portable nebulizer and mask ^2^. In the control group, the pups were treated with saline only. After treatment, pups were euthanized at P3 and P7 for further investigation.

**Tissue preparation**

Pups were sacrificed by an intraperitoneal injection of chloral hydrate and cervical dislocation. A blunt cannula was inserted and tied to the trachea, and the left lobes were inﬂated by instillation of buffered formaldehyde (4% paraformaldehyde solubilized in PBS, pH 7.4) at a pressure of 20 cmH_2_O for 20 min. The left lung was removed and was further ﬁxed in buffered formaldehyde for 24 h at 4°C for subsequent hematoxylin and eosin (H&E) staining and immunohistochemical analysis. The right lung lobe was frozen in liquid nitrogen and stored at -80°C for later measurement of proteins.

**Preparation of NETs *in vitro***

Mouse bone-marrow-derived neutrophils were isolated using a neutrophil isolation kit following a protocol provided by the manufacturer (130-097-658, Miltenyi Biotec). The isolation procedure was conducted at 4°C unless otherwise specified. In brief, mice were anesthetized, and the animal surface was sprayed with 70% ethanol. The muscles were removed from both legs, and the femur was separated from the tibia at the knee joint. The bone marrow cells were flushed using a sterile syringe filled with PBS containing 0.5% endotoxin-free bovine serum albumin (BSA) and 2 mM EDTA (magnetic-activated cell-sorting, MACS) and collected into a 50 ml conical tube through a 70 µm cell strainer. The bone marrow cells were collected by centrifugation at 500 g for 5 minutes at 4°C. Neutrophils from bone marrow cells were negatively selected using a neutrophil magnetic bead isolation kit for sorting granulocytes.

The purified neutrophils (1*10^7^) were seeded into a 10-cm cell culture dish in 10 ml complete culture medium and stimulated with LPS (100 μg/ml) for 4 hours at 37°C. NETs were purified as described previously ^3^. Briefly, the culture medium was gently discarded, and the dish was extensively washed with 4°C precooled PBS to harvest cells and NETs. After centrifugation at 450 g for 10 min at 4°C, the supernatant containing NETs was transferred to a 1.5 ml Eppendorf tube and further centrifuged at 18,000 g for 10 min at 4°C. The pellets containing NETs with 100 μl residue were collected and stored at -80°C. OneDrop^TM^ was used to detect the DNA concentration in the NETs.

**Cell culture**

The murine lung epithelial‑12 (MLE‑12) cell line was obtained from the American Type Culture Collection. Primary alveolar epithelial cells (AEC II) from mice were extracted using 0.1% collagenase, 0.25% trypsin, and DNase I from pulmonary tissues, following the subsequent selection with mouse IgG (36111ES60, Yeasen, China) as described in the literature ^4^. The AEC II and MLE‑12 cells were maintained in high‑glucose Dulbecco's modified Eagle's medium (DMEM; HyClone; GE Healthcare Life Sciences) with 10% fetal bovine serum (FBS; Gibco; Thermo Fisher Scientific, Inc.) in a 5% CO_2_ humidified atmosphere at 37°C.

**Fluorescence microscopy**

The left lung lobe was embedded in OCT (36309ES61, YEASEN, China) at -80°C for at least 24 h. By using a cryostat microtome, the frozen tissue block was cut into 7 μm slices, which were then placed on coated glass slides, fixed with 4% precooled paraformaldehyde for 15 minutes and rehydrated in PBS. After gently washing in PBS three times, the slides were blocked with 5% goat serum (Gibco, 16210-064) for 30 min at 37°C to reduce nonspeciﬁc binding. After blocking, the sections were washed in PBS and stained with anti-histone H3 (citrulline R2 + R8 + R17) (1:300) and anti-MPO antibody (1:50) at 4°C in darkness. After staining, the slides were washed with phosphate buffer saline (PBS), followed by incubation with the secondary antibodies Alexa Fluor 647-anti-mouse IgG (1:500) or Alexa Fluor 555-anti-rabbit IgG (1:500) at 37°C for 1 hour in darkness. After washing in PBS to remove the unbound ﬂuorescent antibody, nuclei in the samples were stained with 4′6-diamidino-2-phenylindole (DAPI). Images were captured by Zeiss LSM7 confocal fluorescence microscopes using the appropriate lenses and filters.

Toll-like receptor 4 (TLR4) expressions in MLE-12 cells were validated with PE-anti-mouse CD284 (TLR4) antibody (1:100) or isotype control antibody. Images were captured by a Zeiss LSM7 confocal fluorescence microscope using the appropriate lenses and filters.

**Protein extraction and western blotting**

Total protein from the cells or tissues was extracted by lysis with RIPA buffer containing protease and phosphatase inhibitor cocktails (Beyotime, Shanghai, China) and was sonicated on ice 3 times for 20 seconds each time. Protein concentrations were determined by a BCA assay ^5^. The samples were separated by 10% SDS-PAGE and transferred to a polyvinylidene diﬂuoride (PVDF) membrane at 300 mA for 90 or 220 min. After blocking for 1 h at room temperature with 5% BSA, the PVDF membranes were incubated overnight at 4°C with primary antibody diluted to the recommended concentration in TBS containing 5% BSA. After washing with TBST three times for 10 minutes each time, goat anti-rabbit HRP IgG (EarthOx Life Sciences, CA, USA) or goat anti-mouse HRP IgG (EarthOx) was used to detect the primary antibodies. After washing with TBST four times for 10 minutes each time, the binding of the speciﬁc antibody was detected with Immobilon Western Chemiluminescent HRP Substrate (Millipore, MA, USA) and visualized using the G:Box gel doc system (Syngene, UK).

**Statistics analysis**

Lung structure was measured as described before ^1, 6^. Statistical analysis was performed using GraphPad Prism 7. Quantitative data were shown as the mean ± standard error of the mean. Data from two groups were compared using the t-test, and data from more than two groups were compared using analysis of variance (ANOVA) with a post hoc Bonferroni test. P < 0.05 was considered to be statistically significant.

**Reference**

1. Li H, Yuan X, Tang J, Zhang Y. Lipopolysaccharide disrupts the directional persistence of alveolar myofibroblast migration through EGF receptor. *Am J Physiol Lung Cell Mol Physiol*;**2012**.*302.*(6):L569-79

2. Cortjens B, et al. Local dornase alfa treatment reduces NETs-induced airway obstruction during severe RSV infection. *Thorax*;**2018**.*73.*(6):578-80

3. Najmeh S, Cools-Lartigue J, Giannias B, Spicer J, Ferri LE. Simplified Human Neutrophil Extracellular Traps (NETs) Isolation and Handling. *J Vis Exp*;**2015**.98)

4. Nabhan AN, Brownfield DG, Harbury PB, Krasnow MA, Desai TJ. Single-cell Wnt signaling niches maintain stemness of alveolar type 2 cells. *Science*;**2018**.*359.*(6380):1118-23

5. Lovrien R, Matulis D. Assays for total protein. *Curr Protoc Microbiol*;**2005**.*Appendix 3.*(Appendix 3A

6. Pua ZJ, et al. Histochemical analyses of altered fetal lung development following single vs multiple courses of antenatal steroids. *J Histochem Cytochem*;**2005**.*53.*(12):1469-79
